# Design and Electrochemical Characterization of Spiral Electrochemical Notification Coupled Electrode (SENCE) Platform for Biosensing Application

**DOI:** 10.3390/mi11030333

**Published:** 2020-03-24

**Authors:** Abha Umesh Sardesai, Vikram Narayanan Dhamu, Anirban Paul, Sriram Muthukumar, Shalini Prasad

**Affiliations:** 1 Department of Bioengineering, The University of Texas at Dallas, Richardson, TX 75080, USA; abha.sardesai@utdallas.edu (A.U.S.); vikramnarayanan.dhamu@utdallas.edu (V.N.D.); anirban.paul@utdallas.edu (A.P.); 2 EnLiSense LLC, 1813 Audubon Pondway, Allen, TX 75013, USA; sriramm@enlisense.com

**Keywords:** concentric design, electrochemical biosensor, C-reactive protein

## Abstract

C-reactive protein (CRP) is considered to be an important biomarker associated with many diseases. During any physiological inflammation, the level of CRP reaches its peak at 48 h, whereas its half-life is around 19 h. Hence, the detection of low-level CRP is an important task for the prognostic management of diseases like cancer, stress, metabolic disorders, cardiovascular diseases, and so on. There are various techniques available in the market to detect low-level CRP like ELISA, Western blot, etc. An electrochemical biosensor is one of the important miniaturized platforms which provides sensitivity along with ease of operation. The most important element of an electrochemical biosensor platform is the electrode which, upon functionalization with a probe, captures the selective antibody–antigen interaction and produces a digital signal in the form of potential/current. Optimization of the electrode design can increase the sensitivity of the sensor by 5–10-fold. Herein, we come up with a new sensor design called the spiral electrochemical notification coupled electrode (SENCE) where the working electrode (WE) is concentric in nature, which shows better response than the market-available standard screen-printed electrode. The sensor is thoroughly characterized using a standard Ferro/Ferri couple. The sensing performance of the fabricated platform is also characterized by the detection of standard H_2_O_2_ using a diffusion-driven technique, and a low detection limit of 15 µM was achieved. Furthermore, we utilized the platform to detect a low level (100 ng/mL) of CRP in synthetic sweat. The manuscript provides emphasis on the design of a sensor that can offer good sensitivity in electrochemical biosensing applications.

## 1. Introduction

C-reactive protein (CRP) is a ring-shaped protein found in blood plasma, whose concentration rises in response to inflammation. Inflammation is the reaction given by the body to recover. The presence of C-reactive protein is proven to be an indicative biomarker of diseases such as cancer [1], stress [2], metabolic disorders [3], diabetes [4], cardiovascular diseases [5], and respiratory diseases [6]. CRP is getting more and more attractive as a popular biomarker for harmful diseases and, hence, detection of this entity is very popular among the scientific community. The amount of CRP present in body fluid can be directly correlated with inflammation or tissue damage. The cause for inflammation can be physiological or pathological. CRP is the reaction given by the body to recover from inflammation-related diseases. Although there is no direct correlation between the severity and amount of CRP in body fluid, it is still considered to be an important marker protein for physiological inflammation. 

During the process of healing from inflammation, CRP reaches its peak value within 48 h. The half-life for CRP is around 19 h [7]. After that, the stimulus is suppressed by increased production of CRP, whereby production is ceased gradually, returning to the base value. This small upregulation in our physiological system lasts for a few hours, and it is very hard to measure levels across an elapsed timeframe. Very often, the methods preferred to measure CRP level are based on immunonephelometric [8] and immunoturbidimetric [9] assays using a single polyclonal antibody and enzyme-linked immunosorbent assay (ELISA) [10]. Although these methods are still popular in the scientific community, there are disadvantages of these methods in that they are expensive and complex in nature, and they require sophisticated instrumentation, more time, and trained personnel. There is still a requirement in the scientific community for biosensors that are field-deployable and easy to use. The literature report suggests various types of sensors varying in the underlying principle of application such as field effect transistor (FET) [11], optical technologies [12], electrochemical methods, and piezoelectric approaches [13]. Each method has its pros and cons. For example, optical technologies are useful for real-time monitoring but there are underlying problems with implementation. Electrochemical methods [14] are highly sensitive with easy implementation, and they are a candidate for miniaturization. Our group explored the electrochemical platform to fabricate a biosensor for important protein markers including CRP [15,16,17,18]. 

Currently, the identification of biomarkers is important for any treatment, and this form of disease management is called prognostic, which involves detecting disease in an early stage. Hence, the detection of CRP in an early stage is necessary to avoid further health issues. Point of care systems (POCs) are very useful in providing rapid detection with a continuous feedback loop. The increased number of smartphone users worldwide fueled intensive research in the sector of POCs [19]. POCs are usually handheld devices or in the form of wearables, which requires sensors to be in miniature form [20]. 

The electrochemical sensing method is currently considered to be the most suitable platform for POCs. The electrochemical sensing platform offers high sensitivity and differentiability, along with the opportunity to deploy in the field. The performance of the sensor is decided by the signal output for the biomarker of interest. The most important component of a sensor assembly is the electrode, as the biochemical reaction occurs at the electrode surface. This makes it essential to look at the parameters of the electrode such as their effective surface area, material, and geometry. In general, the electrochemical sensing platform is made up of electrodes and electrolytes. Generally, electrodes are diversified into different categories based on design, component, and material. Screen-printed three-electrode assemblies with materials like gold, graphene, and carbon are very popular among the scientific community [21]. Bias is applied between the working electrode (WE) and a reference electrode (RE) to get a signature signal output with high sensitivity. This can be achieved by having a counter electrode (CE) in the system, which acts as source/sink for the excess current, helping to reduce the resistance occurring at the WE surface due to polarization.

In the electrochemical sensing platform, three-electrode systems are fundamentally ideal for quantitative monitoring. The electrochemical platform also offers easy transduction to an electrical signal as a function of current/potential. One important factor to consider during the design of the electrochemical sensor is the current distribution between electrodes. Theoretically, it should be spread uniformly over the surface of the electrodes. The use of a CE is fundamentally obtained as a source or sink for the current sources. The presence of a CE makes the measurement of non-faradic current easier despite the presence of faradic current in the system. They can be easily characterized and fine-tuned by adjusting the effective surface area of electrodes. Design parameters are essential for achieving enhanced sensing performance. The fundamental hypothesis behind any electrochemical sensing experiment can be classified into two parts: faradaic and non-faradaic. In both cases, diffusion plays an immense role in current output. Diffusion at the electrode surface is fundamentally understood by Fick’s law, where the concentration gradient is the source of the potential gradient in an electrode electrolyte system. Literature reports suggest that a high surface-to-volume ratio of the electrode can enhance the flux, which is ultimately reflected in the sensing output. Interdigitated electrodes and disc electrodes are examples of such a system where fringes help to increase the effective surface-to-volume ratio. Inspired by such system, we hypothesized a concentric design with maximum efficiency. High selectivity and conductivity in the system are achieved by varying the effective surface area and material used for the WE. The electrochemical sensor platform also gives us the choice of various methods for characterization. Electrochemical impedance spectroscopy (EIS), cyclic voltammetry (CV), and chronoamperometry (CA) are some of the examples currently used in academics, as well as in industry. There are various electrode designs available on the market for different applications. The most famous three-electrode designs are almost identical in shape where the WE is circular in design, and the CE covers three-quarters of the WE as a semicircle, whereas a small square-shaped RE printed with silver/silver chloride is typically present at the end of the semicircle. Scientist tried to increase the surface-to-volume ratio of the WE by modifying the geometry. This generated a newer regime of electrodes called interdigitated electrodes (IDEs) [22]. This kind of electrode increases the effective surface-to-volume ratio, which ultimately increases the conductivity, diffusion, and eventually the signal intensity. For this purpose, IDEs are extensively used in various kinds of biosensing applications. Concentric designs are the newest modeling designs, where fringes provide an increased surface-to-volume ratio. Literature reports suggest that the WE spirality plays an important role in increasing the sensitivity and output current response [23]. Although there are multiple advantages of concentric designs over the bulk electrode design, sharp edges can cause extensive current loss due to the generation of eddy currents. To avoid this loss, sharp edges need to be minimal.

In this paper, we fabricated a concentric gold electrode for its use in standard biosensor applications to detect low levels of CRP. We coined the acronym of this electrode as a spiral electrochemical notification coupled electrode (SENCE). To characterize the sensor platform, we performed standard electrochemical characterization including CV, scan rate variation, EIS, and chrono-coulometry in standard redox electrolyte. We also characterized the sensor in a diffusion environment where we checked the diffusion of H_2_O_2_ toward the electrode surface using chronoamperometry. Finally, we used the platform to detect low levels of CRP in synthetic sweat. A low detection of 100 ng/mL was recorded, which is quite superior in our regard. The ultimate aim of this paper was to validate the concentric design as useful for both faradaic and non-faradaic application in the regime of electrochemical sensing applications. 

## 2. Materials and Methods

The designed SENCE platform was printed over a Flame retardant-4 (FR-4) printed circuit board (PCB), obtained from Vida Bio Technology (Taichung, Taiwan). Potassium ferricyanide (K_3_Fe(CN)_6_), potassium ferrocyanide (K_4_Fe(CN)_6_), potassium chloride (KCl), and hydrogen peroxide (H_2_O_2_) were purchased from Sigma-Aldrich (St. Louis, MO, USA) and were used as received. Deionized (DI) water (18.2 MΩ∙cm) was used for all dilutions. Phosphate-buffered saline (PBS) was procured from Thermo Fisher scientific (Waltham, MA, USA). An artificial sweat formulation (pH 6) was prepared as per the recipe described in previous works [24,25] to be used as the solvent buffer for dose–response studies with the CRP antibody–antigen immunoassay. 

### 2.1. COMSOL

COMSOL Multiphysics (COMSOL INC., Stockholm, Sweden) is a simulation software which allows finite element analysis based on the finite element method (FEM) [26]. It provides an interface where we can define relevant properties for the model such as material properties, constraints, loads, and fluxes, which in turn allows analyzing the behavior of the system. COMSOL uses partial differential equations (PDEs) on every point of the mesh. COMSOL provides an option of choosing the mesh size, which in turn controls the PDE output. It also features adaptive error control techniques using numerical solvers. Along with the mathematical analysis, it provides a visualization tool. This aids in quick gradient-based visualization of the results. In order to build the geometry, it comes with a CAD plugin wherein one can import the CAD (Autodesk Inc., San Rafael, CA, USA) designs and apply the various properties via the material and physical interface. COMSOL supports designs from zero dimensions to three dimensions. COMSOL creates a tree-based structure which starts with the geometry and ends with studies. It iterates through sequencing. Physical constants for the process are predefined. 

During the evaluation of the model, we can have multiple physical phenomena under consideration with the help of various available modules. For this paper, we used the electrochemical module. The electrochemical module basically studies the primary current distribution along with the potential contour. We can define the domain and boundaries as expected in the system, as well as the electrode and electrolyte surface. Optionally, it provides a way to vary the governing equation of the system from the drop-down menu, as well as providing an option to input custom equations based on user-defined parameters. For validation of the electrochemical aspect of the sensor, we used the Cottrell equation and Randall–Sevcik equation. Another aspect of simulating in COMSOL is that it allows us to change the surface properties, such as the porosity of the surface and electrical characteristics of the electrode. It also allows modifying the surface chemistry by allowing an electrode–electrolyte interface. Once the properties are assigned, we can simulate the model.

### 2.2. Design of Sensor

To design the SENCE platform, we chose to have a three-electrode system. The geometry of SENCE consists of a WE, CE, and RE. The substrate for SENCE is FR4. The WE is made of ENIG (electroless nickel immersion gold) on a copper track. The CE is made of carbon, and the RE is made of Ag/AgCl. The most optimized area for WE was found to be 17.13 mm^2^. The areas for the CE and RE were kept constant at approximately 19 mm^2^ and 0.6 mm^2^, respectively. The SENCE platform comes with a 2.5-mm edge pitch connector for easy integration with POCs. The total size of the sensor with the connector is almost 13.2 mm × 25.4 mm. Fundamentally, the current generated in the system is due to the diffusion at the electrode–electrolyte interface. The reason for the diffusion is due to the occurrence of a concentration gradient at the interface where the interface acts as a semi-permeable junction. This diffusion process is governed by Fick’s law. There are two laws defined by the Fick. The first law says the flux of the diffusion is directly proportional to the concentration gradient, as depicted in Equation (1).

(1) $$J=-D\frac{\partial C}{\partial x}$$

where J is the diffusion flux, D is the diffusion coefficient, x is the position, and C is the concentration. 

The second law predicts how diffusion causes the concentration to change with respect to time, as depicted in Equation (2).

(2) $$\frac{\partial C}{\partial t}=D\;\frac{\partial^{2}C}{\partial x^{2}}$$

Our null hypothesis was to simulate the designed geometry using this fundamental equation in COMSOL. The result output should justify the shape and design of the WE to be eligible for the targeted electrochemical application. In this work, we tried to achieve this optimality by changing the shape of the WE to ultimately provide better results in terms of sensitivity.

The original geometry was designed with the CAD plugin, and electrochemical response was analyzed using the COMSOL Multiphysics platform. We used the electrochemical module from COMSOL. Material properties of gold were assigned to the WE. For the CE, we chose the material as carbon, whereas Ag/AgCl was chosen for the RE. After the electrode assignment, we added a block of PBS buffer with pH 7.4 to simulate the electrochemical response. We analyzed the electric potential contour to study the potential distribution. The current distribution profile was checked between the WE and RE and between the WE and CE. Another aspect of the design was the power dissipation profile. The SENCE was also evaluated for its current distribution. All these results are explained in detail in Section 3.

### 2.3. Electrochemical Methods

The custom-made SENCE system was used for all electrochemical experiments. SENCE was designed in AutoCAD and the geometry was optimized in COMSOL before printing on a PCB (printed circuit board). The WE was made of electroless nickel immersion gold (ENIG), whereas the CE was made of carbon and the RE was made of silver/silver chloride (Ag/AgCl). The performance of the custom-made electrode was compared with a standard glassy carbon electrode (GCE) of 3 mm diameter, purchased from CH instrument, and a gold screen-printed DROPSENS electrode, purchased from Metrohm. The GCE was used along with a platinum wire as the CE and Ag/AgCl (saturated KCl) was used as the RE, purchased from CH instruments. The electrochemical experiments were performed in a Gamry series 600 potentiostat/galvanostat. To determine the electrochemical performance of the electrode, standard direct current (DC) techniques were performed with a standard redox couple: Fe(II)/Fe(III). Our aim was to validate the SENCE platform’s electrochemical stability by standardizing it via fitting fundamental electrochemical principles. Cyclic voltammetry was performed in the range of −0.4 V to +0.6 V vs. the Ag/AgCl RE with a scan rate of 25 mV/S. The scan rate was varied from 25 mV/S to 250 mV/S to see the diffusion of electroactive species from the electrolyte to electrode. Chrono-coulometry was performed to obtain the behavior of the electrode, while we performed charge/discharge of redox species at the electrode interface. Chrono-amperometry was performed at a fixed potential of −0.8 V for 30 s. 

As part of this study, non-faradaic EIS was implemented to measure CRP in synthetic sweat (pH 6) to build a label-free biosensor for POC application. All impedance measurements were scanned over a frequency range of 1 Hz to 1 MHz using a Gamry Reference potentiostat (Gamry Instruments, PA, USA) with an alternating current (AC) input of 10 mV and DC bias of −15 mV. The impedance data represented were collected and analyzed for *n* = 3 sensor measures.

### 2.4. Immunoassay Functionalization

Prior to immunoassay functionalization [17], the sensor was thoroughly cleaned with isopropyl alcohol (IPA) and DI water to remove any impurities and then dried using an N_2_ spray-gun. Then, 40 μL of 10 mM dithiobis(succinimidyl propionate) (DSP) (Thermo Fisher Scientific Inc., MA, USA) cross-linker prepared in dimethyl sulfoxide (DMSO) (Thermo Fisher Scientific Inc., MA, USA) was incubated on the sensor chip for 90 min to create a thiol linkage with the electrode surface. *N*-Hydrosuccinimide (NHS)-ester groups present in DSP react with the primary amines of antibodies to form stable amide bonds. Post DSP incubation, a PBS wash step was performed to remove any unbound linker molecules. Then, the sensor was functionalized with 20 μg/mL of capture probe (α-CRP monoclonal antibody) specific to CRP (Abcam, MA, USA) and incubated for 90 min. After antibody conjugation, the sensor surface was washed with PBS to remove any unbound antibody. Next, 40 μL of PBS-based blocking buffer, Superblock (Thermo Fisher Scientific Inc., MA, USA) was added onto the sensor stack and incubated for 15 min to hydrolyze any unbound linker sites, thereby minimizing unspecific binding. Synthetic sweat (pH 6) was then added, and impedance measurements were taken; the artificial sweat formulation with no CRP antigen here acted as a negative control to account for the inherent response of the sensor. CRP concentrations spiked in synthetic sweat (pH 6) were sequentially dispensed on the sensor in increasing order of concentration (from 1 ng/mL to 10ug/mL) and measured at each dose step after 15 min of incubation. The above series of steps were repeated for the SENCE system (number of replicates (*n*) = 3) and a commercially available gold SPE (screen-printed electrode) (*n* = 3) (obtained from Metrohm-Dropsens, USA) to compare performance. The limit of detection (LoD) was determined as the first measurable concentration measured beyond the noise of the system.

## 3. Results and Discussion

### 3.1. COMSOL Results

**Figure 1:**
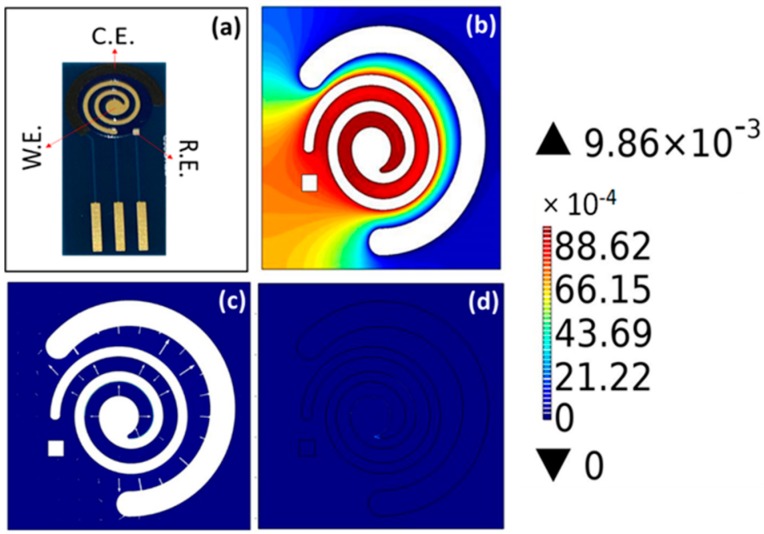
(**a**) Actual image of the spiral electrochemical notification coupled electrode (SENCE) platform. (**b**) Potential contour describing the field distribution. (**c**) Current distribution of the sensor from the working electrode (WE) to the counter electrode (CE) and from the WE to the reference electrode (RE). (**d**) Total power dissipation of the SENCE.

The COMSOL simulation was performed with the drawn design in AutoCAD. Figure 1 explains the results obtained from the COMSOL simulation. This software was used to optimize the parameters for the sensor design. Figure 1a depicts the actual image of the electrode after printing. Figure 1b explains the potential distribution using the contour graph generated. This result is manly governed by the Randles–Sevcik equation described in the electrochemical section. It helps us to understand the field distribution of the sensor when subjected to potential bias. In this case, the value for applied bias was 10 mV. As seen in the contour graph, the electric field was very strong near the WE and gradually decreased toward the CE. Another point to note here is that it remained unaffected by the placement of the RE. Figure 1c explains the current distribution of the sensor. The result suggests that the current was uniformly distributed around the WE, originating from the WE and dispersing toward the CE. Both of these prove the stability of the sensing platform, which remained unaffected by the field effect and hindrance offered by the in-system parameters.

The current profile from the WE to the RE is depicted in Figure S1 (Supplementary Materials), whereas the current from the WE to the CE is depicted in Figure S2 (Supplementary Materials). The main function of RE is to maintain a constant potential. This allows perfect capture of signal at the WE during the electrochemical reaction. The WE undergoes changes in the presence of electrolyte and gives rise to current, which should be concentrated near the WE area. Similarly, if we see the current flow from the WE to the CE, we can observe that it has a peak current near the WE and decreases toward the CE. To get the results, we defined the cut line from WE toward the CE. Moreover, the smooth graph shows that the system was immune to the noise in the system and captured the signal more accurately. One of the reasons behind such a smooth response is actually hidden in the spiral design. The raw dimensions of the spiral design are depicted in Figure S3 (Supplementary Materials). We kept in mind the generation of unintentional eddy currents due to sharp edges and, hence, we wiped out all the possible sharp edges, which helped us to achieve better simulation results. This design modification is also reflected in the electrochemical output, which is discussed later. 

Figure 1d explains the total power dissipation of the SENCE. The system was designed to be power-efficient. COMSOL Multiphysics provides a means to measure the system’s total power dissipation, visualized through the color gradient scheme. The color gradient around the WE is higher in potential, reducing toward the CE and RE. The overall power dissipation of the sensor is much lower.

### 3.2. Electrochemical Characterization of SENCE System

**Figure 2:**
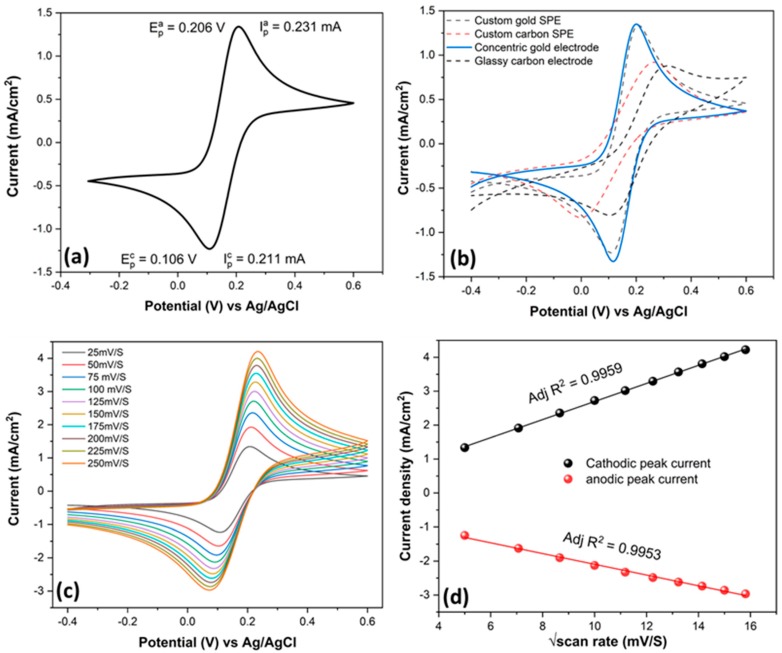
(**a**) Cyclic voltammetry (CV) result of SENCE in 10 mM Fe(II)/Fe(III) diluted in 0.1 M KCl. (**b**) CV comparison of fabricated electrode and commercial electrode available on the market showing the superior result for SENCE. (**c)** Scan rate study using SENCE platform. (**d**) Peak current plotted with scan rate to validate Randles–Sevcik equation, showing linearity in both cathodic and anodic peak current.

The design and fabrication of SENCE led us to characterize its electrochemical stability. To use the sensor for further application, we characterized its electrochemical properties in a standard ferrocyanide/ferricyanide solution, where 10 mM K_3_Fe(CN)_6_ and K_4_Fe(CN)_6_ were diluted in 0.1 M KCl, used as the supporting electrolyte. Cyclic voltammetry was performed in a range of −0.4 to +0.6 V, and the results are depicted in Figure 2a.

The results show the excellent faradaic response of the bare SENCE platform toward the electron transfer of the ferro/ferri couple. A sharp anodic peak appeared at +0.206 V, whereas a sharp cathodic peak was obtained at +0.106 V. The peak separation between cathodic and anodic peaks was exactly 100 mV, which suggests the high electrochemical stability of the SENCE platform toward the redox couple. Ferro/ferri is a renowned redox couple used as a primary standard for any faradaic electrochemical reaction. Any electrochemical reaction is termed as faradaic when the output current polarizes due to the electron transfer originated from redox molecules or couples. As the ferro/ferri couple possesses a single electron transfer, the CV output reflects the polarization of current with two distinctive sharp peaks separated between 80 and 100mV. Such an electrochemical reaction is also termed a reversible electrochemical reaction. The charge originated at the electrolyte diffusing from the bulk to the electrode surface causes the polarization in current and, hence, a sharp peak with excellent peak separation suggests that electron transfer at the fabricated SENCE platform is extremely feasible.

We compared the results of our SENCE platform with conventional electrodes available on the market, and the results are depicted in Figure 2b. Although the electrochemical stability of conventional carbon electrodes is not even close to that of our platform, the bulk gold design showed similar activity to the SENCE platform. However, the effective surface-to-volume ratio of the bulk gold electrode is somewhat smaller than that of the SENCE platform and, hence, the peak current was slightly lower in both anodic and cathodic regions. The kinetics of a redox couple reveals much information about the electrochemical robustness, as well as stability, of an electrode. The Randles–Sevcik equation (Equation (3)) is one of the prime electrochemical hypotheses where electro-kinetic properties come into play.

(3) $$i_{p}=0.4463nFAC{\left(\frac{nF\vartheta D}{\mathrm{RT}}\right)}^{\frac{1}{2}}$$

where *i_p_* is the current maximum in amps, *n* is the number of electrons transferred in the redox event, *A* is the electrode area in cm^2^, F is the Faraday constant in coulombmol^−1^, *D* is the diffusion coefficient in cm^2^/s, *C* is the concentration in mol/cm^3^, ν is the scan rate in V/s, R is the gas constant in J∙K^−1^∙mol^−1^, and T is the temperature in Kelvin.

The equation suggests that the peak current of an ideal faradaic redox couple is directly proportional to the scan rate. To check the electro-kinetic property, we varied the scan rate from 25 to 250 mV/S and plotted the current density versus root mean square of the scan rate. The result is depicted in Figure 2c. The SENCE platform seems extremely stable when we plot the anodic and cathodic peak current. We plotted both the anodic and the cathodic peak current varying with scan rate, and the result is depicted in Figure 2d. The result shows the excellent linearity of both cathodic (adjusted *R*^2^ = 0.9953) and anodic peak current (adjusted *R*^2^ = 0.9959), which increase with increasing scan rate. The electrochemical output of the SENCE platform suggests the excellent stability of the sensor platform for faradaic application, especially for sensing. 

**Figure 3:**
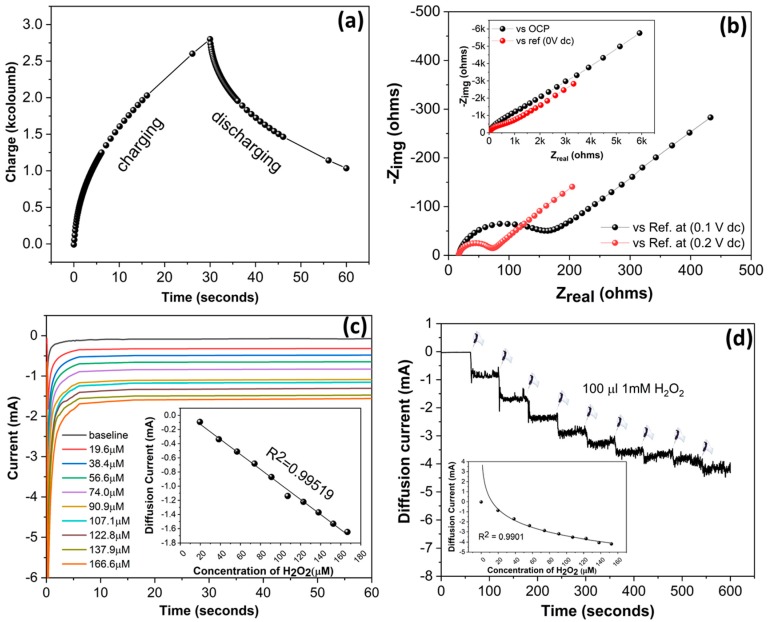
(**a**) Chrono-coulometric single-cycle characterization of SENCE showing proper charging and discharging behavior in a redox environment. (**b**) Electrochemical impedance spectroscopy (EIS) Nyquist behavior of SENCE with a direct current (DC) bias of 0.1 V and 0.2 V showing proper charge transfer. The same experiment in an open circuit and without bias is depicted in the inset. (**c**) Chrono-amperometric sensing of H_2_O_2_ along with calibrated dose response (inset). (**d**) Continuous sensing of H_2_O_2_ along with calibrated dose response (inset).

Keeping this aspect in mind, we performed chrono-colometry using the same ferro/ferri redox couple to see the behavior of the SENCE platform in external charging/discharging. One cycle of charging and discharging was performed as depicted in Figure 3a. The result shows the excellent charging/discharging ability of the fabricated platform in a standard redox system. The inherent conductivity of the metal surface allows the charge to diffuse in its surface, and the inert gold surfaces do not allow the charges to remain; hence, all charges come back during discharging. The experiment provides a green signal to proceed further in utilizing the SENCE platform for application in faradaic sensing experiments.

Electrochemical impedance spectroscopy (EIS) is a powerful tool to estimate the charge transfer at the electrode–electrolyte interface. AC current is applied through the electrode to bias it, and the resulting charge transfer resistance is measured through a Nyquist plot. The experiment was performed using the standard ferro/ferri couple by biasing with a DC bias of 0.1 V and 0.2 V. The result is depicted in Figure 3b. The result depicts a semicircle, implying that the charge transfer resistance decreased at a DC bias of 0.2 V. The reason behind this is that the CV result suggests that the oxidation of ferro to ferri occurs at 0.2 V, which shows charge transfer behavior in the Nyquist plot. The result also suggests the stability of the fabricated sensor under AC pulse. To check that the electron transfer phenomenon is indeed working, we performed the EIS in an open circuit and upon applying 0 V DC. The result is depicted in the inset of Figure 3b. The result shows the absence of a semicircle, which is obvious due to the non-availability of electrons generated from the ferro/ferri couple being transferred either in an open circuit or at 0 V DC. This suggests the robustness of the SENCE platform to respond in the presence and absence of a redox couple. We also varied the pH of the ferro/ferri solution and performed CV. The result is depicted in Figure S4 (Supplementary Materials). The differences in peak potential and peak current were estimated and plotted versus pH. The result is depicted in Figure S5 (Supplementary Materials). The result suggests the obvious distribution of peak potential and current at low pH. An excess of H^+^ should prohibit the Fe(II)/Fe(III) from diffusing toward the electrode surface, resulting in a low peak current and high peak potential separation, which can be seen clearly in the result. The result suggests that the platform is extremely sensitive toward pH. The result also suggests the robustness of the platform toward pH as we increased the pH toward preferred physiological conditions, whereby the peak separation under faradaic regime supports its use in biosensor applications, which are mostly operated at physiological pH. 

These preliminary tests led us to perform standard H_2_O_2_ sensing at the electrode interface. For this purpose, 1 mM H_2_O_2_ was added to a blank solution of 0.1 M KCl, and chronoamperometry was recorded at −0.8 V. Th reason behind choosing chronoamperometry to measure the current was due to the reduction of H_2_O_2_ through a standard diffusive pathway. The result is depicted in Figure 3c where we varied the concentration of H_2_O_2_ from 20 µM to 160 µM and measured the current due to diffusion at a fixed potential of −0.8 V. The phenomenon is governed by the famous Cottrell equation (Equation (4)), where diffusion current is directly proportional to the concentration of the diffused species.

(4) $$i=\frac{nFAc_{j}^{0}\sqrt{D_{j}}}{\sqrt{\pi t}}$$

where, *i* is the current due to diffusion, $c_{j}^{0}$ is the concentration of the diffused species, and *t* is time.

The calibrated dose response is depicted in the inset of Figure 3c, which shows excellent linearity (adjusted *R*^2^ = 0.9959) of the diffusion current while varying the concentration. The result suggests that the SENCE platform is extremely stable in physiological conditions and supports the diffusion of species to its surface to get reduced/oxidized. Continuous sensing is an interesting additive of any sensor platform, where sensors are tested to perform in more robust way. In this kind, the sensor is monitored for a certain time, whereas analyte concentration is varied in a fixed time interval to get a staircase-like response. We repeated the same H_2_O_2_ sensing by adding 100 µL of 1 mM H_2_O_2_ at a time interval of 60 s and for a total period of 600 s. The data are depicted in Figure 3d. The calibrated dose response was calculated, and the result is plotted in the inset of Figure 3d, which suggests that the sensor response is surprisingly excellent in supporting diffusion to its surface. The concentric nature of the electrode draws more diffusion due to capacitive charge build-up at the electrode surface. All of these standard electrochemical characterizations prove the SENCE platform to be useful for any kind of standard faradaic sensing. The results also influenced us to use the platform for a non-faradaic environment where a biosensor assay was tested and found to be superior in this fabricated platform.

### 3.3. Electrochemical Characterization of the Immunoassay Using Electrochemical Impedance Spectroscopy

**Figure 4:**
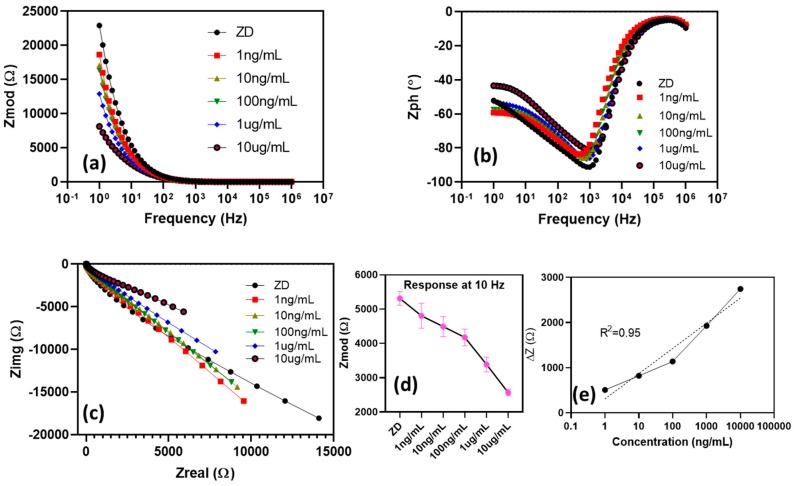
(**a**) Bode magnitude plot representing impedance measurement at every C-reactive protein (CRP) concentration spiked in synthetic sweat (pH 6). (**b**) Bode phase plot for impedance measured corresponding to CRP concentration in synthetic sweat (pH 6). (**c**) Nyquist plot representing change in impedance of CRP in synthetic sweat (pH 6). (**d**) Impedance response at 10 Hz measured at every CRP concentration in synthetic sweat (pH 6). (**e**) Semi-log fit, i.e., log of concentration on *x*-axis and change in impedance from baseline on *y*-axis, for *n* = 3 replicates.

An open-circuit potential (OCP) test was performed to assess electrode stability, as well as the inherent potential of the sensor system. Here, OCP is the potential difference measured between the WE and RE under no applied current. Typically, open-circuit measurements lie in the range of 10^−3^ V [27], and the OCP of the developed SENCE platform was determined as −15 mV. Hence, the SENCE electrode is viable for use as an electrochemical biosensor. The electrochemical performance of the SENCE platform was studied using a label-free EIS method for the detection of CRP in synthetic sweat. EIS was widely characterized in use for biosensor applications, and it was proven to be highly sensitive to the electrochemical interactions at the functionalized electrode surface [28,29]. To prove applicability of the SENCE platform in biosensor point-of-care systems, the specific binding interaction of the CRP biomarker was tested in a bio-fluid mimic buffe of -synthetic sweat (SS) (pH 6) by measuring the impedance response starting from the lowest concentration to the highest concentration for the specific biomarker. The impedance results can be plotted as Nyquist and Bode plots for SS spiked with CRP in the range from 1 ng/mL to 10 ug/mL, as shown in Figure 4. The measurements were taken over a frequency spectrum of 1 Hz to 1 MHz for *n* = 3 replicates for both the SENCE platform and the commercially available gold SPE.

For a non-faradaic EIS system, there is no electron transfer due to the absence of a redox tag (label-free). Hence, in the Nyquist plot, effects due to charge transfer resistance (R_ct_) and Warburg impedance (Z_w_) become infinite and, hence, are not visible in the curve. A representative result of this non-faradaic impedance analysis is shown by the large incomplete semicircle due to the absence of the electron transfer step and diffusion tail [30]. Therefore, in this case, the imaginary part of impedance (Z_img_) is inversely related to the electrical double layer capacitance. Furthermore, the binding of the CRP molecules to the captured antibody creates a charge modulation at the surface of the electrode due to perturbation in the dielectric permittivity [31] thereby causing a capacitive change to the electrical double layer (EDL) associated with a typical affinity-based system. This is observed in Figure 4c, where an increase in CRP dose corresponds to a decrease in the imaginary impedance values. Therefore, this decrease in impedance can be attributed to the increase in CRP protein concentration. The capacitive nature of this system can be further observed in the Bode magnitude and phase plots in Figure 4a,b. Furthermore, the maximum signal-to-noise ratio (SNR) was evaluated at 10 Hz, and the result was selected to analyze the calibrated dose response of CRP in SS (pH 6), as shown in Figure 4d. The change in impedance (Z_mod_) from baseline at 10 Hz varied from around 500 Ω to 3000 Ω with an *R*^2^ of 0.95 (*y* = 242.59ln(*x*) + 311.12) (Figure 4e), and a detection limit of 100 ng/mL was found, determined as the dose above noise level that can be clearly distinguishable (three standard deviations of the baseline). The low AC potential of 10 mV used in this technique helps to polarizes the electrode interface, thereby causing a change in impedance due to the modulation of charge. Hence, the dose-dependent change in impedance noted here occurs as a function of capacitive build-up with the binding of antibody and CRP (antigen). The sensor performance proved that the dynamic range of the CRP assay in SS (pH 6) lies within the physiologically relevant ranges for serum [17].

**Figure 5:**
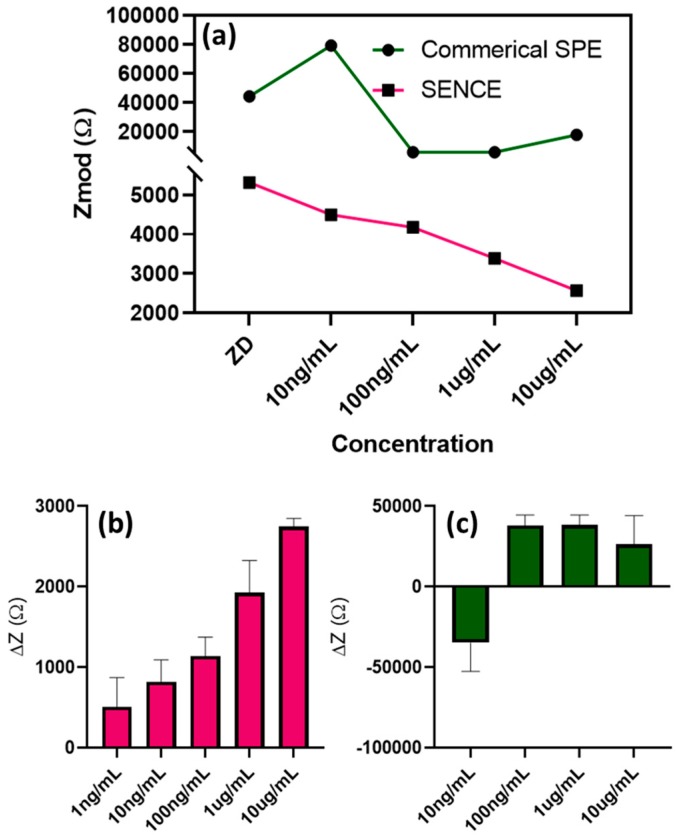
(**a**) Comparison plot of impedance responses at 10 Hz between SENCE platform (this work) and a commercially available screen-printed electrode (SPE). (**b**) Calibration dose response of CRP in synthetic sweat (pH 6) represented as the change in impedance from baseline in the SENCE platform. (**c**) Calibration dose response of CRP in synthetic sweat (pH 6) represented as the change in impedance from baseline in the commercial SPE.

The change in impedance plot with respect to baseline is plotted for SENCE in Figure 5b against that of the commercially available SPE in Figure 5c. This experiment was performed to compare the response of the developed SENCE platform. From the non-linearity of delta *Z* (CDR plots), it was determined that the SPE was not responsive to the developed functionalized assay for CRP optimized for the SENCE platform. This non-linear trend of the response in the gold SPE compared with our design (Figure 5a) cements the merits of the SENCE system as a biosensor in this proof-of-applicability study.

## 4. Conclusions

In this study, we designed a new biosensing platform named SENCE by utilizing the concentric design of a WE. The design was drawn in AutoCAD and simulated in COMSOL. The sensor was characterized thoroughly from an electrochemical point of view. CV, scan rate variation, chrono-coulometry, and faradaic EIS measurements were performed using a ferro/ferri redox couple to obtain the electrochemical stability of the sensor. Chrono-amperometry was performed to obtain the standard H_2_O_2_ sensing. The results support the fact that the sensor platform supports diffusion from electrolyte to electrode. Finally, we utilized the sensor for low-level detection of CRP in synthetic sweat. The non-faradaic EIS technique was implemented, and a 100 ng/mL detection limit was obtained. The result was compared to a standard screen-printed electrode, and the SENCE system was found to be superior. The unique design, along with the sensitivity and electrochemical stability of SENCE, can be an excellent alternative to commercial electrodes available on the market.

## Supplementary Materials

The following are available online at https://www.mdpi.com/2072-666X/11/3/333/s1: Figure S1: The current profile from working electrode to reference electrode; Figure S2: The current profile from working electrode to counter electrode; Figure S3: Raw design of the Spiral electrochemical notification coupled electrode; Figure S4: Cyclic voltammetry of Fe(II)/Fe(III) in different pH, showing the sensor is very much stable in pH variation; Figure S5: Plot of peak potential separation and peak current difference showing the sensor is very sensitive towards pH.
