# Supplementary material for: Design and Electrochemical Characterization of Spiral Electrochemical Notification Coupled Electrode (SENCE) Platform for Biosensing Application
Source: Micromachines (Basel). 2020 Mar 24;11(3):333. doi: 10.3390/mi11030333 (PMC7143249; doi:10.3390/mi11030333)
Supplement: Supplementary file 1 [file micromachines-11-00333-s001.pdf]

micromachines

Supplementary Materials:

Design and Electrochemical Characterization of Spiral Electrochemical Notification Coupled Electrode (SENCE) Platform for Biosensing Application

Abha Umesh Sardesai [1,†], Vikram Narayanan Dhamu [1,†], Anirban Paul [1], Sriram Muthukumar [1,2] and Shalini Prasad [1,2,*]

[1] Department of Bioengineering, The University of Texas at Dallas, Richardson, Texas 75080, USA; abha.sardesai@utdallas.edu (A.U.S.); vikramnarayanan.dhamu@utdallas.edu (V.N.D.); anirban.paul@utdallas.edu (A.P.)
[2] EnLiSense LLC, 1813 Audubon Pondway, Allen, TX 75013, USA; sriramm@enlisense.com
* Correspondence: shalini.prasad@utdallas.edu
† These authors contributed equally to this work.

Received: 2 March 2020; Accepted: 23 March 2020; Published: date

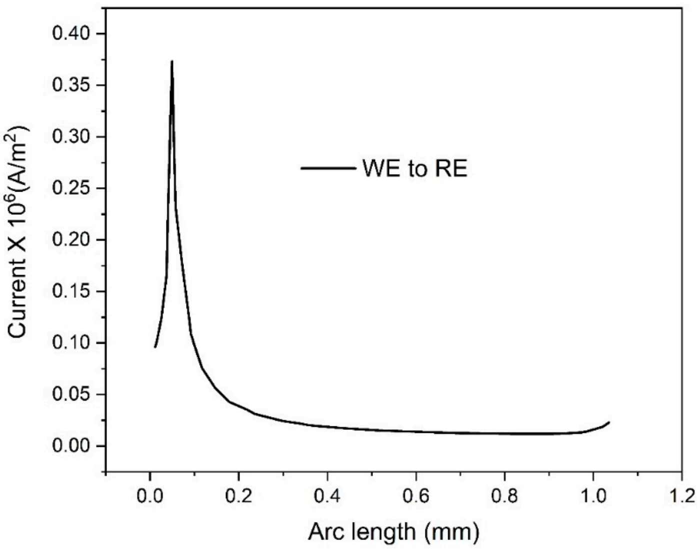

Figure 1. The current profile from working electrode to reference electrode. WE: working electrode; RE: reference electrode.

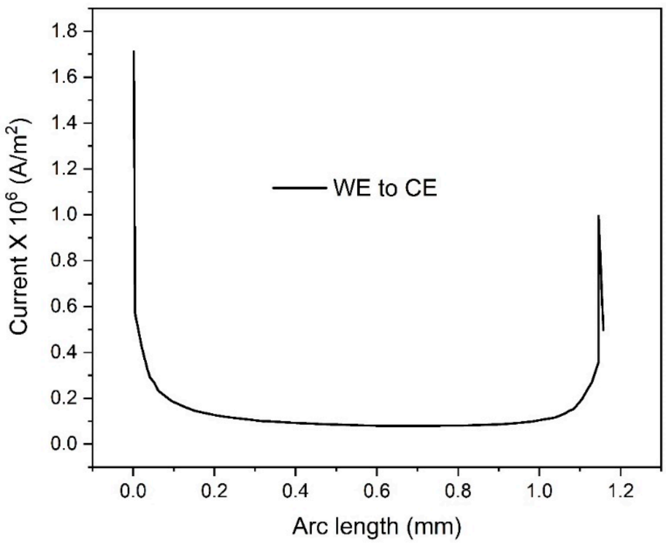

Figure S2. The current profile from working electrode to counter electrode. CE: counter electrode.

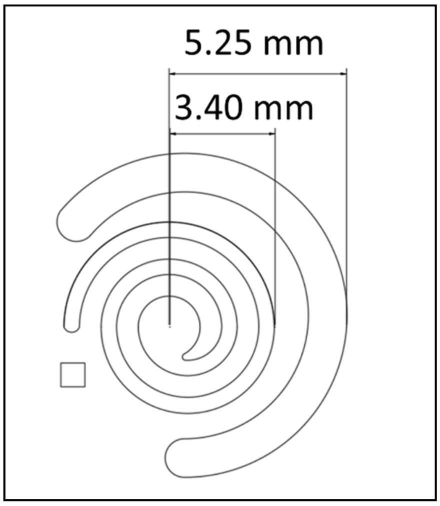

Figure S3. Raw design of the Spiral electrochemical notification coupled electrode.

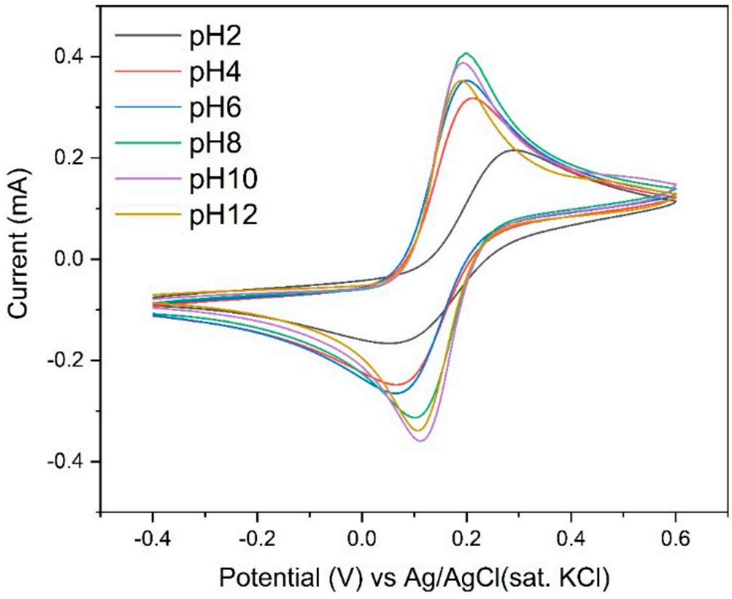

Figure S4. Cyclic voltammetry of Fe(II)/Fe(III) in different pH, showing the sensor is very much stable in pH variation.

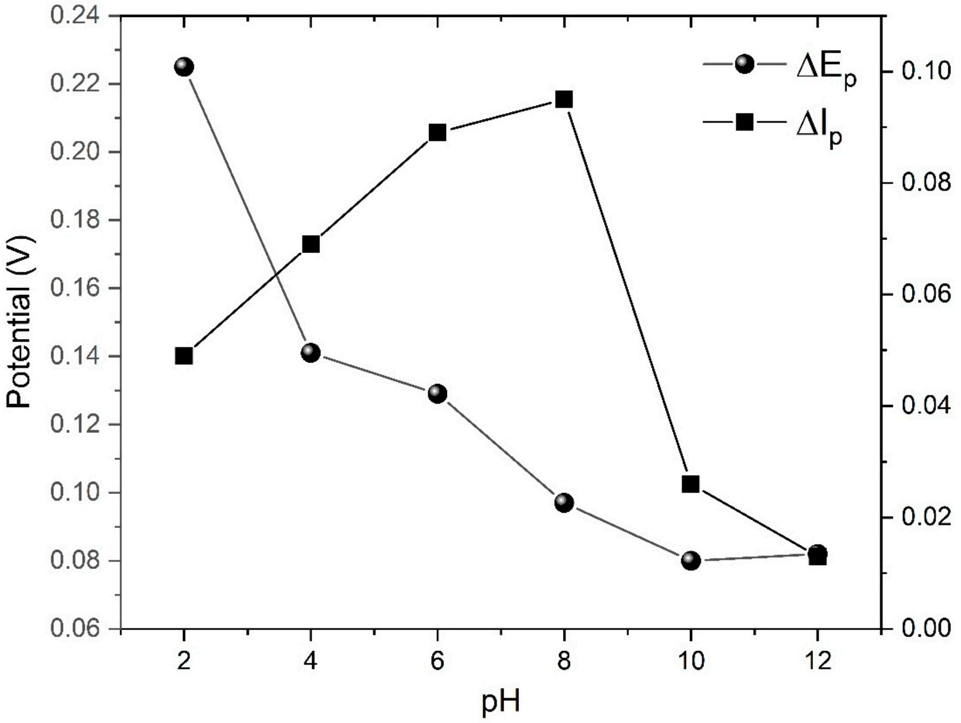

Figure S5. Plot of peak potential separation and peak current difference showing the sensor is very sensitive towards pH.

© 2020 by the authors. Submitted for possible open access publication under the terms and conditions of the Creative Commons Attribution (CC BY) license (http://creativecommons.org/licenses/by/4.0/).
